# *In situ* preparation of Ag nanoparticles on silicon wafer as highly sensitive SERS substrate

**DOI:** 10.1039/c7ra12955f

**Published:** 2018-01-12

**Authors:** Xinglong Tu, Zheng Li, Jing Lu, Yanpeng Zhang, Guilin Yin, Weiming Wang, Dannong He

**Affiliations:** School of Material Science and Engineering, Shanghai Jiao Tong University No. 800 Dongchuan Road Shanghai 200240 PR China hdn_nercn@163.com +86-21-3429-1125 +86-21-3429-1286; National Engineering Research Center for Nanotechnology 200241 Shanghai China jinglu2004@163.com; School of Mechanical Engineering, Shanghai Jiao Tong University No. 800 Dongchuan Road Shanghai 200240 PR China

## Abstract

An intensive surface enhanced Raman scattering (SERS) effect is realized by ordered Ag nanoparticles (NPs) *in situ* grown on silicon wafer directly using (3-aminopropyl) trimethoxysilane (APS) as both the surface modifier and reducing agent. The as-prepared ordered Ag NPs based SERS substrate shows excellent performance in detecting glycerin (an important integration in liquid super lubricating system) as well as conventional Rhodamine 6G (R6G, a kind of dye organic pollutant). The enhancement factor (EF) achieves 4-fold for glycerin and 10-fold for R6G (allowing for detecting as low as 10^−11^ M aqueous R6G), confirming the high sensitivity. The limited relative standard deviations (RSD) of the enhancement factors are within 15% for both glycerin and R6G, indicating the excellent uniformity. This remarkable progress is ascribed to the advantages of APS in improving adsorption and modulating distribution of Ag NPs on silicon, which results in a large local electric field to enhance the Raman signals. The SEM and UV-visible absorption spectrum characterization verified the contribution of APS in SERS improvement by investigating the influence of APS content and reduction time during the preparation process. All these advances imply that the SERS substrates prepared by Ag NPs *in situ* grown on silicon wafer have great potential application in real-time interface state tracing and sensitive detection.

## Abstract 2

An intensive surface enhanced Raman scattering (SERS) effect is realized by ordered Ag nanoparticles (NPs) *in situ* grown on silicon wafer directly using (3-aminopropyl) trimethoxysilane (APS) as both the surface modifier and reducing agent.

## Graphical abstract

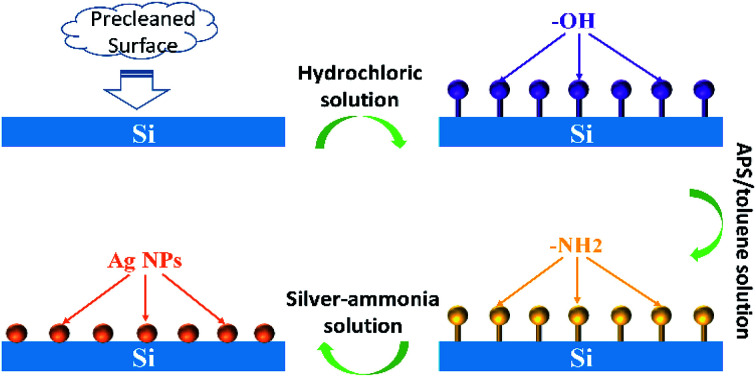

## Introduction

Surface enhancement Raman scattering (SERS) has attracted much attention in various fields since it was firstly reported in 1974,^1^ such as environmental monitoring, analytical chemistry and biological medicine due to its unique characters including high sensitivity, quick response, noninvasive analysis and fingerprint recognition.^2–7^ Consequently, tremendous studies have been carried out on exploring different kinds of highly sensitive SERS substrates. In these reports, noble metal NPs,^8–10^ semiconductor substrates^11–13^ and graphene^14–17^ have all been used in SERS measurement.

Among the developed SERS substrates mentioned above, noble metal NPs, especially Ag NPs based SERS substrates, aroused special interest owing to their higher enhancement of Raman signals.^18–23^ However, most Ag NPs based SERS substrates could be categorized as either Ag NPs in colloid solution or Ag NPs fabricated on solid substrates. Compared with colloid solution, the latter kind has shown better performance in improvement of large-area uniformity for efficient modulation of Ag NPs distribution. Thus, great efforts have been made to prepare Ag NPs on solid substrates in various approaches, including electron-beam lithography,^19^ thermal decomposition,^20^ immersion plating^21^ chemical reduction.^18,22,23^ However, all these fabrication methods are too complex to modulate features of nanoscale Ag NPs, such as the shape, size, quantity, and distribution, which play a critical role in achieving best SERS performance. Moreover, easy aggregation and loose bond of Ag NPs with the solid substrate has always been a big drawback.^24,25^

In this study, we introduce a novel approach to *in situ* prepare Ag NPs on Si wafer as highly sensitive SERS substrate by employing APS as both the surface modifier and reducing agent, which contributes a lot to generation and distribution modulation of Ag NPs. The as-prepared SERS substrate shows high sensitivity and excellent uniformity. In particular, it exhibits excellent performance in detecting glycerin, which plays an important role in liquid super lubricating system.^26–28^ Besides, aqueous R6G, which is a kind of dye organic pollutants,^29–31^ can be detected with a concentration low as 10^−11^ M. Furthermore, the limited standard deviation of the enhancement factors of both glycerin and R6G presents good uniformity in random spot detection process. Scanning electron microscope (SEM) and UV-visible absorption spectroscopy characterizations during the reduction process are carried out to explore the influence of APS on preparation of Ag NPs, confirming its contribution in modulating the distribution of Ag NPs. All these results indicate this *in situ* preparation of Ag NPs on Si wafer a novel approach to fabricate highly sensitive SERS substrate, which may open a new window may open a new window to trace the real-time friction interface state and clarify the mechanism behind the liquid super lubricating system. Moreover, a brief explanation of the enhancement mechanism was given in preparation process discussion.

## Experimental

### Reagents and materials

Silicon wafers were purchased from Sinopharm Chemical Reagent Co., Ltd (China). APS, silver nitrate (>99%), toluene, aqueous ammonia solution (28%) and ethanol were all supplied by Sinopharm Chemical Reagent Co., Ltd (China). Deionized water with resistance of 15 MΩ from Milli-Q system was used throughout the experiment.

### *In situ* preparation of Ag nanoparticles on silicon wafer

Silicon plates (2 × 2 cm^2^) were immersed in hydrochloric acid and boiled for 2 h and then left to cool at room temperature in the acidic solution. After this, the plates were rinsed with distilled water and dried for 1 h at 100 °C to complete the cleaning process. These cleaned plates were immersed in toluene (95%) solutions of APS at 90 °C under vigorous stirring with 24 h. The initial volume ratio of APS/toluene were set as 1/10, 3/10, 5/10, 7/10, and 9/10. After this, the wafers were rinsed with a copious amount of methanol to remove extensive absorbed APS. Then the rinsed wafers were treated ultrasonically in deionized water and left to dry for 1 h at 100 °C. Noticeable, removing excessive APS from silicon surface was of great importance in preventing aggregation of Ag NPs in the next step.

Then, the modified silicon wafers were immersed in silver–ammonia solution at 30 °C for 24–96 h under vigorously stirring. The dispersed Ag NPs would *in situ* grow on silicon wafer, whose size and distribution were modulated by the concentration of the silver–ammonia solution in a range of 0.1–1.5 M.

### Characterization of Ag NPs and SERS performance measurement

Field-emission scanning electron microscope (FE-SEM, S-4800, Hitachi, Japan) was used to observe the surface morphology and nanostructure of Ag NPs. UV-visible spectrometer (Perkin Elmer: Lambda2) performed the absorbance characterization of prepared Ag NPs. The atom information of the prepared SERS substrates was determined by an Energy Dispersive Spectroscope (EDS, ORAN System SIX). The SERS performance was evaluated through confocal microscope Raman system (Renishaw: Invia-reflex) with a 532 nm diode laser and 1800 lines per mm grating observed through a 50× LWD objective.

## Results and discussion

**Fig. 1:**
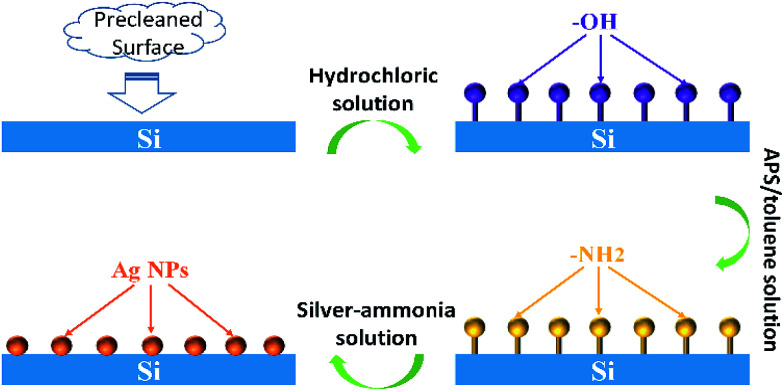
Scheme of Ag NPs *in situ* prepared on silicon wafers using APS as both surface modifier and reducing agent.

The Ag NPs were *in situ* fabricated on the silicon wafers with proper modification by APS. The scheme of this process is shown as Fig. 1. The pre-treated silicon plates were easily amine-functionalized due to the condensation reaction between hydroxyl on silicon surface and methoxy group of APS, resulting in a strong adhesion.^32^ Then the amine function acted as reducing agent to generate Ag NPs and attach them. The size, amount and distribution of Ag NPs depend on the concentration of APS and silver–ammonia solution as well as reaction time. With optimization, the silicon substrate with Ag NPs attached presents high SERS activity that can significantly enhance the Raman signal intensity of the probe molecules mentioned above.

**Fig. 2:**
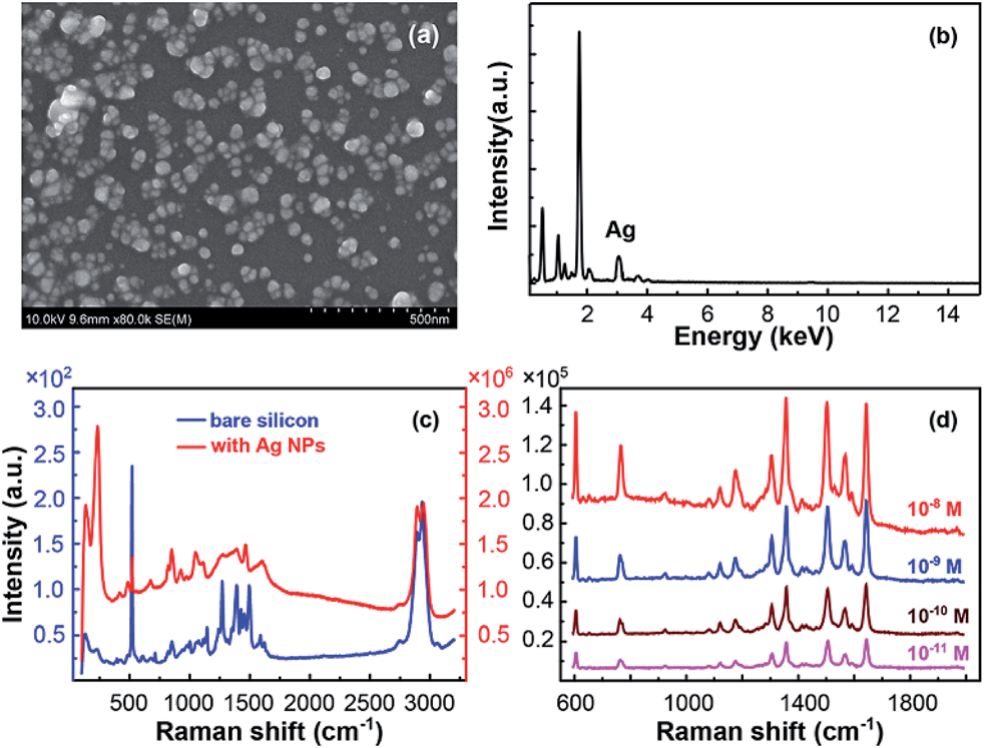
(a) SEM and (b) EDS characterization of the optimized SERS substrate with the best performance. The performance of the as-prepared SERS substrate using (c) glycerin and (d) R6G as probe molecule.

The SEM and EDS characterization of the SERS substrate based on Ag NPs with best performance were exhibited as Fig. 2(a) and (b), which has been optimized by fabrication process modulation. The optimized fabrication condition was as follows. Firstly, silicon wafer was modified by APS/toluene solution at volume ratio of 9/10. Then the modified wafer was immersed in 1.1 M silver–ammonia solution for 96 h. After this, Ag NPs were generated and well distributed on silicon surface.

To present the excellent performance of this optimized SERS substrates, we demonstrated the Raman spectra of pure glycerin on silicon without and with Ag NPs in Fig. 2(c). The high sensitivity of the as-prepared substrate for R6G is exhibited by detecting low concentration solution, shown in Fig. 2(d). The results indicate this *in situ* preparation of Ag nanoparticles on silicon wafer substrate a high sensitive SERS substrate, which has excellent capability to enhance the Raman signals for both molecules. The EF of pure glycerin signal, calculated according to the equation EF = (*I*_s_ × *C*_0_)/(*C*_s_ × *I*_0_),^33^ achieves 4-fold enhancement which is the highest until now. Here, *I*_s_ and *I*_0_ are peak intensities of the band at 1451 cm^−1^ for glycerin absorbed on the optimized SERS substrate and bare silicon wafer, respectively. For R6G molecule, the signal could be clearly detected even when the concentration was diluted to low as 10^−11^ M, which corresponds to an EF of 4.9 × 10^10^ calculated in the same method. For both glycerin and R6G, this optimized SERS substrate exhibited competitive sensitivity among noble metal NPs based SERS substrates.^23,34,35^

**Fig. 3:**
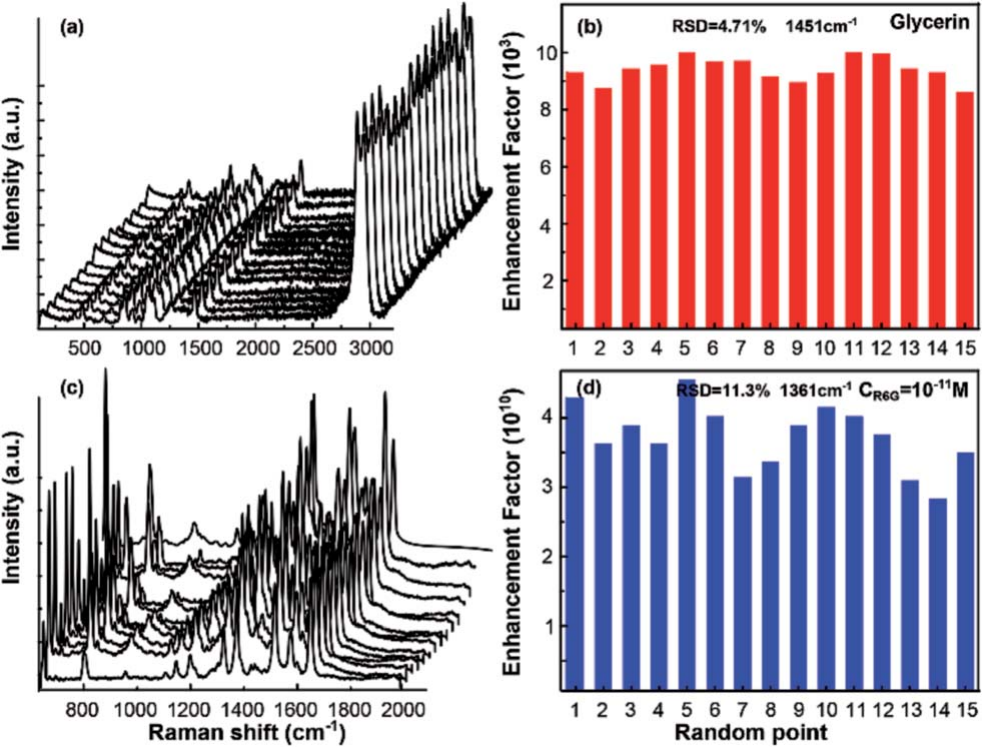
The Raman spectra obtained from 15 random points on the optimized substrate for (a) pure glycerin and (c) R6G solution at 10^−11^ M; RSD of the EF obtained from the 15 random spots for (b) pure glycerin and (d) R6G.

In Fig. 3, the uniformity was also exhibited to further confirm the performance of the as-prepared SERS substrate. As is shown in Fig. 3(a) and (c), the Raman spectra obtained from 15 random spots were demonstrated. Each spot displayed distinctive Raman intensity for glycerin and R6G, implying the outstanding reproducibility of the SERS substrate. The relative standard deviations (RSDs) of EF obtained from these 15 random spots, shown as Fig. 3(b) and (d), was 4.71% for glycerin and 11.3% for R6G (much less than 20%) separately, which certified the high uniformity of the SERS performance.^34^

**Fig. 4:**
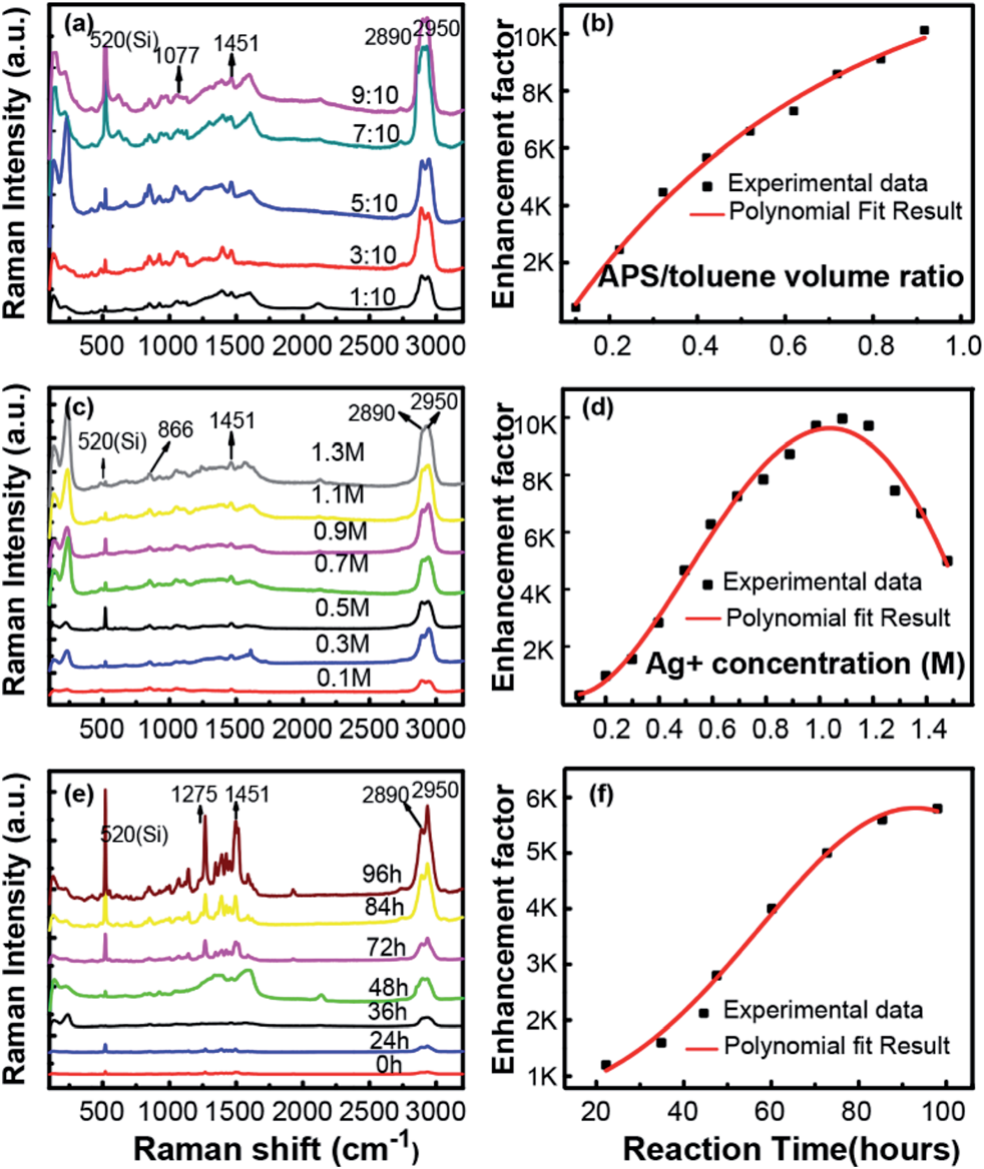
The Raman spectra of glycerin on the prepared substrate in different conditions and the dependence of EF on corresponding factors (a and b) APS/toluene volume ratio (c and d) silver ammonia solution concentration (e and f) reduction time.

All above analysis implies that this *in situ* preparation of Ag NPs on silicon wafer a novel approach to fabricate highly sensitive SERS substrate. To illustrate the influence of fabrication process on this superior SERS performance, a series of experiments were carried out to verify the contribution of those key factors, including the APS and silver–ammonia solution concentrations as well as the reaction time. The performance of SERS substrates prepared in different conditions are exhibited Fig. 4, employing glycerin as probe molecule.

As is shown in Fig. 4(a), the Raman spectra observed with different volume ratio of APS/toluene solution were presented, with a constant silver–ammonia solution concentration of 1.1 M and a reduction time of 96 h. The intensity of the Raman signals became stronger and stronger with the increasing APS volume ratio. The dependence of EF on APS volume ratio was exhibited as Fig. 4(b). It was clearly seen that the EF eventually increased and finally reached the highest of 4 times with the increasing APS/toluene volume ratio. This increasing enhancement was attributed to more function of APS modified on the silicon surface, which would generate more Ag NPs (more hot spots) *in situ* on silicon surface. Then larger local electric fields would be generated in adjacent Ag NPs and enhanced the Raman excitation of the detecting molecule more. Noticeable, we use a 9/10 volume ratio of APS/toluene as a threshold value due to the uncertain performance of a higher one (corresponding EF changes in a wide range), resulting from too much APS layers stack on silicon surface. The dependence of SERS performance on the silver–ammonia solution concentration (Ag^+^ concentration) is illustrated in Fig. 4(c) and (d), the intensity of the Raman signals kept increasing with silver–ammonia solution concentration until 1.1 M. Then the EF would decrease a little if the concentration continued to increase. This increasing EF was also ascribed to more attached Ag NPs produced with a higher Ag^+^ concentration. However, once the concentration exceeded the threshold value, those excessive Ag^+^ ions produced Ag NPs would lead to an aggregation. This would in turn influence the plasmon coupling between adjacent Ag NPs and result in a decreased EF. Here the APS volume ratio was kept constant at 9/10 and the reduction time was kept 96 h. The corresponding dependence of the Raman signal on reduction time was given in Fig. 4(e) and (f). The intensity of the Raman signals and EF kept increasing at first and tended to be steady after 96 h. During the reduction process, the Ag NPs were generated firstly and gradually grew due to a complete reaction. Here the maximum of EF was about 6000 due to a limited silver–ammonia solution concentration (0.9 M).

**Fig. 5:**
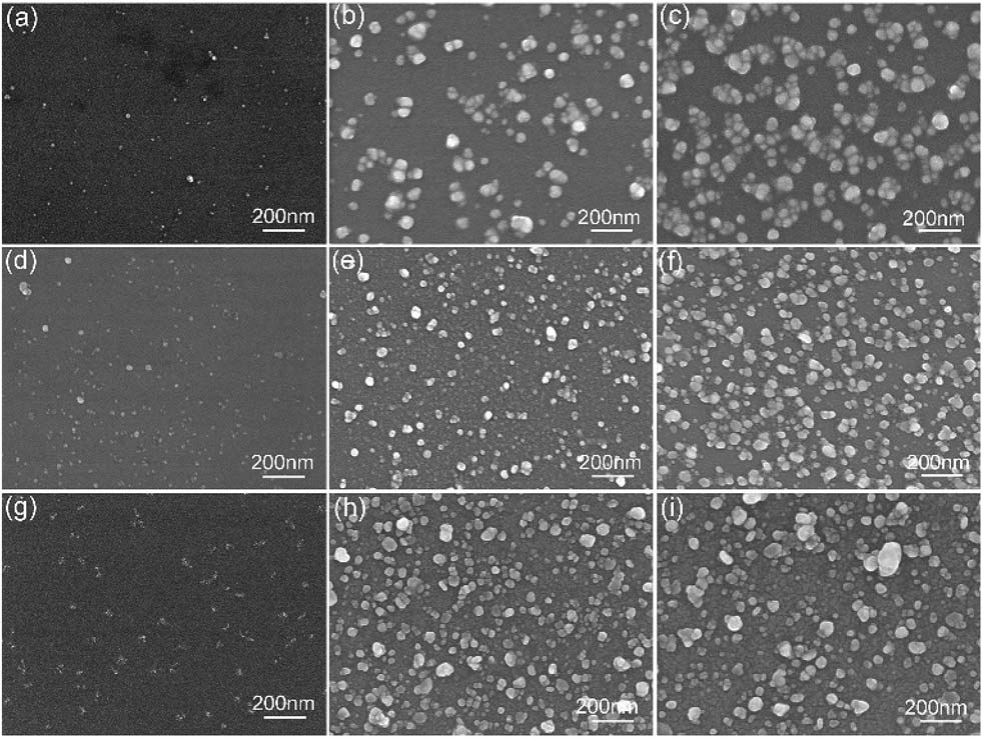
SEM images of Ag NPs *in situ* grow on silicon wafers in different conditions (a–c) volume ratio of APS/toluene solutions were 1/10, 5/10 and 9/10 with 1.1 M silver–ammonia solution and 96 h reduction time (d–f) silver–ammonia solution concentration is 0.1 M, 0.5 M and 0.9 M with 9/10 APS/toluene volume ratio and 96 h reduction time (g–i) reduction time is 24 h, 48 h and 72 h with 9/10 APS/toluene volume ratio and 1.3 M silver–ammonia solution.

To further verify the influence of this *in situ* preparation process on the size, amount and distribution of Ag NPs, the SEM images of Ag NPs prepared in different condition were displayed as Fig. 5. According to the SEM characterization, a higher APS or silver–ammonia solution concentration could lead to more Ag NPs generation with more uniform distribution. The size depended on the reduction time more. All this contributed to a stronger enhancement as Fig. 4 showed. The silicon modified by a higher concentration of the APS contributed to more Ag nucleation attached firstly. With an increasing silver–ammonia solution concentration and a longer reduction time, the Ag nucleation grew *in situ* larger and became more dispersed on the silicon surface as Ag NPs. The formation and distribution of Ag NPs played key roles in SERS performance,^36^ which was also identical with the results shown in Fig. 4.

**Fig. 6:**
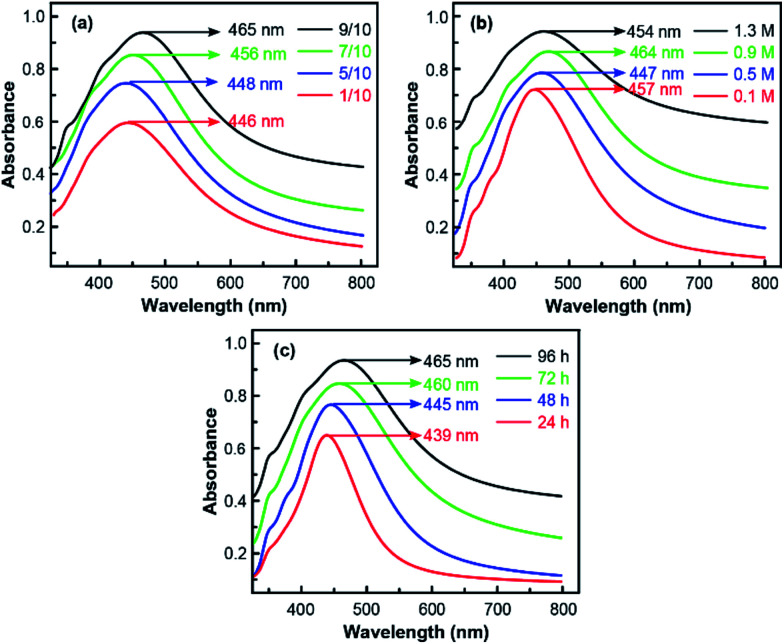
Comparison of UV-visible absorption spectrum of *in situ* prepared Ag NPs on silicon wafer in different (a) APS/toluene volume ratio with a 1.1 M silver–ammonia solution and 96 h reduction time (b) silver–ammonia solution concentrations with a 9/10 APS/toluene volume ratio and 96 h reduction time (c) reduction time with a 1.1 M silver–ammonia solution and 9/10 APS/toluene volume ratio.

In Fig. 6, UV-visible absorption spectrum results were given to further certify the mechanism behind the excellent SERS performance. As is shown, the absorption peak observed around 450 nm confirmed the formation of Ag NPs on silanised silicon wafers. Besides, more detailed information could be obtained during the *in situ* preparation process from the results. In Fig. 6(a) and (b), the intensity of absorption peak substantially increased with APS/toluene volume rate as well as silver–ammonia solution concentrations due to more Ag NPs attached on the Si substrate, which was in consist with the results of SEM images. However, with more Ag NPs produced, the size of generated Ag NPs distributed in a wider range and induced a more broadened absorption band.^36,37^ The red shift of absorption peak (towards long wavelength) in Fig. 6(a) and (b) also indicated larger Ag NPs generated during the preparation process. By contrast, the influence of reduction time was more directly. As displayed in Fig. 6(c), both the broadened absorption band and red shift of peak were induced by a longer reaction time. In other words, more and larger Ag NPs were generated with a full reaction. Thus, the optimized SERS substrate could be obtained by modulating the size and distribution of Ag NPs *in situ* preparation process.

## Conclusions

In summary, a highly sensitive SERS substrate was developed by *in situ* preparing Ag nanoparticles on silicon wafer in a novel approach. Using APS as both surface modifier and reducing agent contributed a lot to controlling the size and distribution of Ag NPs during the preparation process. The optimized as-prepared SERS substrate exhibited competitive sensitivity and high uniformity in enhancing both the glycerin and R6G signals. The Raman signal of glycerin exhibited 4-fold enhancement on the optimal substrate, while the detection limit for R6G reached 10^−11^ M. Besides, the RSD of EF in random spot scanning was much less than conventional Ag NPs based SERS substrates. All results indicated that *in situ* fabrication of Ag NPs on silicon wafer could service as a versatile SERS substrate with high sensitivity and uniformity. This may open a new window for clarifying the mechanism behind the liquid super lubricating system through tracing real-time interface state.

## Conflicts of interest

There are no conflicts of interest to declare.

## Supplementary Material

## References

[cit1] Fleischmann M., Hendra P. J., McQuillan A. J. (1974). Chem. Phys. Lett..

[cit2] Cao Y. C., Jin R., Mirkin C. A. (2002). Science.

[cit3] Kelly K. L., Coronado E., Zhao L. L., Schatz G. C. (2003). J. Phys. Chem. B.

[cit4] Kvitek L., Prucek R., Panacek A., Novotny R., Hrbac J., Zboril R. (2005). J. Mater. Chem..

[cit5] Ding L.-P., Fang Y. (2007). Appl. Surf. Sci..

[cit6] Abu Hatab N. A., Oran J. M., Sepaniak M. J. (2008). ACS Nano.

[cit7] Matta C., Joly-Pottuz L., De Barros Bouchet M. I., Martin J. M., Kano M., Zhang Q., Goddard W. A. (2008). Phys. Rev. B.

[cit8] Fang Y., Wei H., Hao F., Nordlander P., Xu H. (2009). Nano Lett..

[cit9] Ruey-Chi W., Yong-Siang G., Shu-Jen C. (2009). Nanotechnology.

[cit10] Smythe E. J., Dickey M. D., Bao J., Whitesides G. M., Capasso F. (2009). Nano Lett..

[cit11] Ye W., Shen C., Tian J., Wang C., Hui C., Gao H. (2009). Solid State Sci..

[cit12] Li X., Chen G., Yang L., Jin Z., Liu J. (2010). Adv. Funct. Mater..

[cit13] Yang L., Ma L., Chen G., Liu J., Tian Z.-Q. (2010). Chem.–Eur. J..

[cit14] Chen A., DePrince A. E., Demortière A., Joshi-Imre A., Shevchenko E. V., Gray S. K., Welp U., Vlasko-Vlasov V. K. (2011). Small.

[cit15] Fan M., Andrade G. F. S., Brolo A. G. (2011). Anal. Chim. Acta.

[cit16] Ma Z.-Z., Zhang C.-H., Luo J.-B., Lu X.-C., Wen S.-Z. (2011). Chin. Phys. Lett..

[cit17] Que R., Shao M., Zhuo S., Wen C., Wang S., Lee S.-T. (2011). Adv. Funct. Mater..

[cit18] Wang C., Fang J., Jin Y., Cheng M. (2011). Appl. Surf. Sci..

[cit19] Xia N., Cai Y., Jiang T., Yao J. (2011). Carbohydr. Polym..

[cit20] Liang W., Chen X., Sa Y., Feng Y., Wang Y., Lin W. (2012). Appl. Phys. A.

[cit21] Shao Q., Que R., Shao M., Cheng L., Lee S.-T. (2012). Adv. Funct. Mater..

[cit22] Wang Y. Q., Ma S., Yang Q. Q., Li X. J. (2012). Appl. Surf. Sci..

[cit23] Ye Y., Liu H., Yang L., Liu J. (2012). Nanoscale.

[cit24] Zhou Y., Chen J., Zhang L., Yang L. (2012). Eur. J. Inorg. Chem..

[cit25] Li J., Zhang C., Ma L., Liu Y., Luo J. (2013). Langmuir.

[cit26] Xu W., Mao N., Zhang J. (2013). Small.

[cit27] Zhang L. (2013). Appl. Surf. Sci..

[cit28] Liu C.-S., Li B.-H., Chen C.-H., Peng J.-W., Lee S. (2014). J. Raman Spectrosc..

[cit29] Yang S., Dai X., Stogin B. B., Wong T.-S. (2016). Proc. Natl. Acad. Sci. U. S. A..

[cit30] Rajoriya S., Bargole S., Saharan V. K. (2017). Ultrason. Sonochem..

[cit31] Shen K., Gondal M. A. (2017). J. Saudi Chem. Soc..

[cit32] Kango S., Kalia S., Celli A., Njuguna J., Habibi Y., Kumar R. (2013). Prog. Polym. Sci..

[cit33] Kang L., Chu J., Zhao H., Xu P., Sun M. (2015). J. Mater. Chem. C.

[cit34] Kurouski D., Van Duyne R. P. (2015). Anal. Chem..

[cit35] Liu X., Wang J., Wu Y., Fan T., Xu Y., Tang L., Ying Y. (2015). Sci. Rep..

[cit36] Yang L., Qin X., Jiang X., Gong M., Yin D., Zhang Y., Zhao B. (2015). Phys. Chem. Chem. Phys..

[cit37] Milekhin A. G., Sveshnikova L. L., Duda T. A., Yeryukov N. A., Rodyakina E. E., Gutakovskii A. K., Batsanov S. A., Latyshev A. V., Zahn D. R. T. (2016). Phys. E.

